# λ stenting: a novel technique for posterior communicating artery aneurysms with fetal-type posterior communicating artery originating from the aneurysm dome

**DOI:** 10.1007/s00234-021-02775-y

**Published:** 2021-08-05

**Authors:** Jun Tanabe, Ichiro Nakahara, Shoji Matsumoto, Yoshio Suyama, Jun Morioka, Akiko Hasebe, Sadayoshi Watanabe, Kenichiro Suyama, Kiyonori Kuwahara, Keiko Irie

**Affiliations:** 1 Department of Comprehensive Strokology, Fujita Health University School of Medicine, 1-98 Dengakugakubo, Kutsukake-cho, Toyoake, Aichi Japan; 2 Department of Neurosurgery, Nagoya Kyoritsu Hospital, 1-172 Hokke, Nakagawa-ku, Nagoya, Aichi Japan

**Keywords:** Aneurysm, Posterior communicating artery, Endovascular, Coiling, Stent

## Abstract

**Purpose:**

Endovascular treatment of posterior communicating artery aneurysms with fetal-type posterior communicating artery originating from the aneurysm dome is often challenging because, with conventional techniques, dense packing of aneurysms for posterior communicating artery preservation is difficult; moreover, flow-diversion devices are reportedly less effective. Herein, we describe a novel method called the λ stenting technique that involves deploying stents into the internal carotid artery and posterior communicating artery.

**Methods:**

Between January 2018 and September 2020, the λ stenting technique was performed to treat eight consecutive cases of aneurysms. All target aneurysms had a wide neck (dome/neck ratio < 2), a fetal-type posterior communicating artery with hypoplastic P1, and a posterior communicating artery originating from the aneurysm dome. The origin of the posterior communicating artery from the aneurysm, relative to the internal carotid artery, was steep (< 90°: V shape).

**Results:**

The maximum aneurysm size was 8.0 ± 1.9 mm (6–12 mm). The average packing density (excluding one regrowth case) was 32.7 ± 4.2% (26.8–39.1%). Initial occlusion was complete occlusion in 6 (75.0%) patients and neck remnants in 2 (25.0%) patients. Follow-up angiography was performed at 18.4 ± 11.6 months (3–38 months). There were no perioperative complications or reinterventions required during the study period.

**Conclusion:**

The λ stenting technique enabled dense coil packing and preservation of the posterior communicating artery. This technique enabled safe and stable coil embolization. Thus, it could become an alternative treatment option for this sub-type of intracranial aneurysms.

**Supplementary Information:**

The online version contains supplementary material available at 10.1007/s00234-021-02775-y.

## Introduction

Posterior communicating artery (PCOM) aneurysms constitute approximately 30% of all aneurysms [1]. Endovascular therapy remains the first-line treatment, especially for ruptured or posterior circulation aneurysms [2, 3]. Moreover, many PCOM aneurysms are treated safely and effectively by endovascular coiling. Although a fetal-type PCOM itself is not a risk factor, PCOM aneurysms with a fetal-type PCOM “originating from the aneurysm dome” are a risk factor for initial incomplete embolization and long-term instability [4, 5]. Dense packing of the aneurysm and preservation of the PCOM is difficult using a conventional stent and/or balloon-assisted adjunctive techniques. Furthermore, flow diversion devices have been reported to be less effective for the treatment of fetal-type PCOM aneurysms [6, 7]. However, drastic advancements in endovascular therapy have enabled stent deployment in the PCOM. Herein, we describe a novel method called “the λ stenting technique” that involves deploying stents to the internal carotid artery (ICA) and the PCOM, thereby creating a lambdoid-shaped stent to achieve a relatively dense packing of aneurysms and successful preservation of the PCOM.

## Methods

### Clinical application

The present study is a retrospective review of medical charts and radiographic data. The study protocol was conducted in accordance with the principles of the Declaration of Helsinki and approved by the institutional ethics committee. The need for written informed consent for participation was waived in accordance with the national legislation and institutional requirements. On our institute’s website, all participants were provided with the opportunity to opt out of this study. Decisions for radical treatment modalities were made jointly by neurological and endovascular surgeons. Between January 2018 and September 2020, the λ stenting technique was performed to treat eight consecutive cases of aneurysms. The criteria to target aneurysm in the present study were as follows: all target aneurysms had wide neck (dome/neck ratio < 2), a fetal-type PCOM with hypoplastic P1, a PCOM caliber > the P1 segment of the posterior cerebral artery, and a PCOM originating from the aneurysm dome. The PCOM origin from the aneurysm, relative to the ICA, was steep (< 90°: V shape). The aneurysm volume was calculated using a 3D-rotational angiography workstation (Canon Medical Systems, Japan; Philips Medical Systems, the Netherlands). The coil volume was calculated using the following equation:

$$\mathrm{Coil volume }=\uppi \times {(\mathrm{diameter of coil}/2)}^{2}\times \mathrm{length of coil}$$

The packing density was calculated as the ratio of the coil volume to the aneurysm volume.

### Technical details

All procedures were performed under general anesthesia. Perioperative dual antiplatelet management (aspirin, 100 mg/day; clopidogrel, 75 mg/day) was administered at least 14 days before the procedure. The period of postoperative antiplatelet therapy depended on the follow-up angiographic result and the discretion of the attending physician. In general, the dual antiplatelet therapy was maintained for 6 months after the procedure; a single agent was prescribed indefinitely thereafter. Systemic heparinization was maintained with an activated clotting time of at least 250 s during the procedure.

**Fig. 1:**
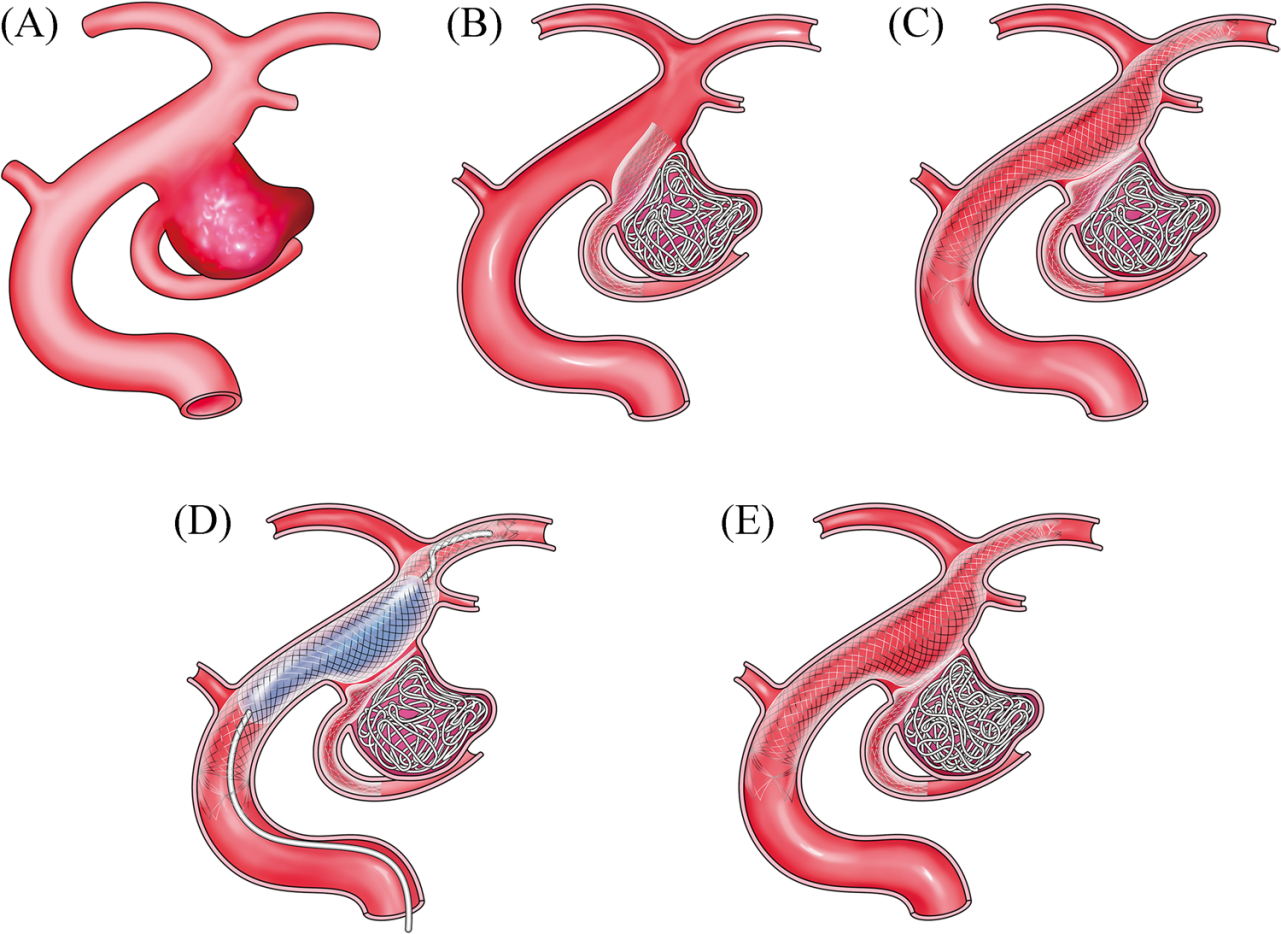
Schematic image of the λ stenting technique used for fetal-type PCOM aneurysms (**A**). A low-profile stent is deployed in the PCOM after insertion of as many coils as possible using the balloon assisted technique (**B**). A braided stent is deployed in the ICA, covering the neck of the aneurysm (**C**). In-stent percutaneous transluminal angioplasty is performed to acquire complete stent apposition; the PCOM stent is crushed in the aneurysm neck between the coil mesh and the ICA stent (**D**). The ICA and PCOM stents form a complex stent in the lambda configuration, namely ‘the λ stent’ (**E**). PCOM, posterior communicating artery; ICA, internal carotid artery

Technical details of the λ stenting technique are presented in Fig. 1. A large-bore guiding catheter (an 8-Fr guiding catheter or a 7-Fr guiding sheath), which accepts three microcatheter systems, was placed at the cervical internal carotid artery. The tip of a pre-shaped microcatheter, such as the Excelsior SL10 J in most cases or the C pre-shaped microcatheter (Stryker, Kalamazoo, MN, USA), or a manually shaped microcatheter, such as a micro pigtail, was directed toward the orifice of the PCOM antegradely. A steerable but flexible micro guide wire with a soft tip, such as Traxcess 14 (MicroVention-Terumo, Tustin, CA, USA), GT wire (MicroVention-Terumo), or Tenrou 1014 (Kaneka Medix, Kanagawa, Japan), was advanced up to the P3 or to a more distal position in the posterior cerebral artery. Thereafter, the microcatheter followed the micro guide wire deeply into the PCOM. Before stent deployment, coils were previously inserted through another microcatheter with balloon assistance (mostly Scepter C; 4.0 mm in diameter, 15 mm in length; Microvention-Terumo) and catheter assistance of PCOM, while avoiding the protrusion of coils over the ICA and PCOM, respectively. The following stenting procedures are essential to this novel technique. A low-profile short-length open-cell stent (Neuroform Atlas; 3.0 mm in diameter, 15 mm in length; Stryker) was deployed from the PCOM to a point slightly distal to the aneurysmal neck, to open the distal flair forward distal neck (Fig. 1B). Subsequently, a braided stent, such as the LVIS (MicroVention-Terumo), was deployed through Headway 21 (exchanged from the balloon catheter or the PCOM microcatheter) in the ICA covering the aneurysm neck using the bulging technique (Fig. 1C). In-stent percutaneous transluminal angioplasty was performed, where necessary, using Scepter or another compliant balloon for complete stent apposition (Fig. 1D). Consequently, the ICA stent crushed a part of the PCOM stent that was placed at the aneurysm neck between the ICA stent and coil mass, creating a complex stent shaped in a lambda configuration, termed “λ stenting” (Fig. 1E). Thus, the neck was covered with one layer of the ICA stent and two layers of the PCOM stent, leading to high metal coverage almost equal to the flow diverter at the aneurysm neck. Finally, coil embolization was advanced for a high packing density of the aneurysm, where possible.

### Clinical and radiological assessments

Digital subtraction angiography (DSA) or magnetic resonance angiography (MRA) was recommended at 3, 6, and 12 months after coil embolization. Angiographic results were evaluated with the modified Raymond-Roy occlusion classification on DSA or MRA [8]. As the neck of the target aneurysm in the present study was obscure, we considered the neck of these aneurysms as the border between the PCOM stent and the aneurysm wall. Therefore, aneurysms with no filling over PCOM stenting were defined as a modified Raymond-Roy occlusion classification of 1. Perioperative neurological outcomes were evaluated by reviewing the hospital records. Perioperative complications were defined as a deterioration of one point or more in the preoperative modified Rankin scale due to endovascular therapy.

## Results

Eight patients underwent λ stenting (Table 1): three men and five women, with a mean age of 63.6 ± 13.6 years (45–85 years). Among them, six patients presented with pure unruptured aneurysms, and two patients had regrowth of their aneurysm after previous coiling and clipping, following aneurysm rupture. The maximum aneurysm size was 8.0 ± 1.9 mm (6–12 mm). Successful initial occlusion was achieved in all cases: 6 (75.0%) patients had complete occlusion with a modified Raymond-Roy occlusion classification of 1 and 2 (25.0%) patients had neck remnants with a modified Raymond-Roy occlusion classification of 2. The average packing density (excluding one case of aneurysm regrowth) was 32.7 ± 4.2% (26.8–39.1%). Furthermore, the average procedure times were 170 ± 50 min (100–270 min). Follow-up angiography was performed at 18.4 ± 11.6 months (3–38 months). Follow-up angiographic results remained unchanged in all patients. None of the patients experienced perioperative complications or required reintervention during the study period. Dual antiplatelet therapy was maintained at 5.6 ± 2.8 months (3–12 months). Thereafter, a single agent was maintained in all patients except 1 case: patient 2 for whom the administration of a single agent was discontinued 3 years after the procedure.

**Table 1:** Patients characteristics

Patient	Age	Sex	Aneurysm diameter (mm)	Symptoms	Postoperative result by modified RROC	Packing density (%)	Procedure times (min)	Perioperative complications	Follow-up angiography (month)	Reintervention	DAPT period (month)
1	60	M	12 × 6 × 6	Regrowth	1	NA	210	None	3	None	3
2	68	F	8 × 7 × 7	None	2	29.6	150	None	38	None	6
3	45	F	7 × 6 × 6	None	1	30.1	200	None	18	None	6
4	85	F	6 × 5 × 4	None	1	33.9	100	None	34	None	6
5	53	F	7 × 5 × 4	None	1	39.1	120	None	20	None	6
6	79	F	10 × 8 × 7	None	2	26.8	270	None	12	None	3
7	48	M	6 × 5 × 5	None	1	38.2	120	None	16	None	12
8	71	M	8 × 5 × 5	Regrowth	1	31.4	190	None	6	None	3

*DAPT*, dual antiplatelet therapy; *NA*, not available; *RROC*, Raymond-Roy occlusion classification

Table 2 shows the technical details of λ stenting in each case. In all cases, the transfemoral approach was used. Balloon assistance in the ICA distal to the neck was employed in 3 (37.5%) cases to navigate the microcatheter for the PCOM. A pre-shaped microcatheter (PCOM catheter) was used for PCOM cannulation in 7 (87.5%) cases. In one case (patient 3), a manually shaped microcatheter into a micro pigtail was used. The Neuroform Atlas was utilized for the PCOM stent in all cases. LVIS was utilized for the ICA stent in all cases except one. The in-stent percutaneous transluminal angioplasty was performed using Scepter or SHOURYU (Kaneka Medix, Osaka, Japan) in 6 (75.0%) cases.

**Table 2:** Technical details of λ stenting

Patient	Guiding catheter	Assist for PCOM cannulation	Balloon catheter	PCOM catheter	PCOM stent	ICA stent	In stent PTA
1	8-Fr Roadmaster	None	Scepter C 4 × 15	SL10 J pre-shaped	NF 4.5 × 15	LVIS 4.5 × 18	Scepter C 4 × 15
2	7-Fr Shuttle sheath	Half deployment of ICA stent and coils of aneurysm	Scepter C 4 × 15	SL10 J pre-shaped	NF 3 × 15	LVIS 4.5 × 23	Scepter C 4 × 15
3	8-Fr Roadmaster	None	Scepter XC 4 × 11	Headway17 micro pigtail manual shaped	NF 3 × 15	LVIS 4 × 22	SHOURYU HR 7 × 7
4	8-Fr Roadmaster	Balloon assist	Scepter C 4 × 15	SL10 C pre-shaped	NF 3 × 15	NF 4.5 × 21	none
5	8-Fr Roadmaster	None	Scepter C 4 × 15	SL10 J pre-shaped	NF 3 × 15	LVIS 4.5 × 23	Scepter C 4 × 15
6	8-Fr Roadmaster	None	Scepter C 4 × 15	SL10 J pre-shaped	NF 3 × 15	LVIS 4 × 22	none
7	8-Fr Roadmaster	Balloon assist	Scepter C 4 × 15	SL10 J pre-shaped	NF 3 × 15	LVIS 4 × 17	SHOURYU HR 7 × 7
8	8-Fr Roadmaster	Balloon assist	Scepter XC 4 × 11	SL10 J pre-shaped	NF 3 × 15	LVIS 4.5 × 23	Scepter XC 4 × 11

*PCOM*, posterior communicating artery; *ICA*, internal carotid artery; *NF*, Neuroform Atlas, SL 10 (Stryker); *PTA*, percutaneous transluminal angioplasty; Headway 17, Scepter C and XC, LVIS (Terumo); Roadmaster (Goodman); Shuttle sheath (Cook Medical); SHOURYU HR (Kaneka Medix)

### Representative cases

#### Case 1

**Fig. 2:**
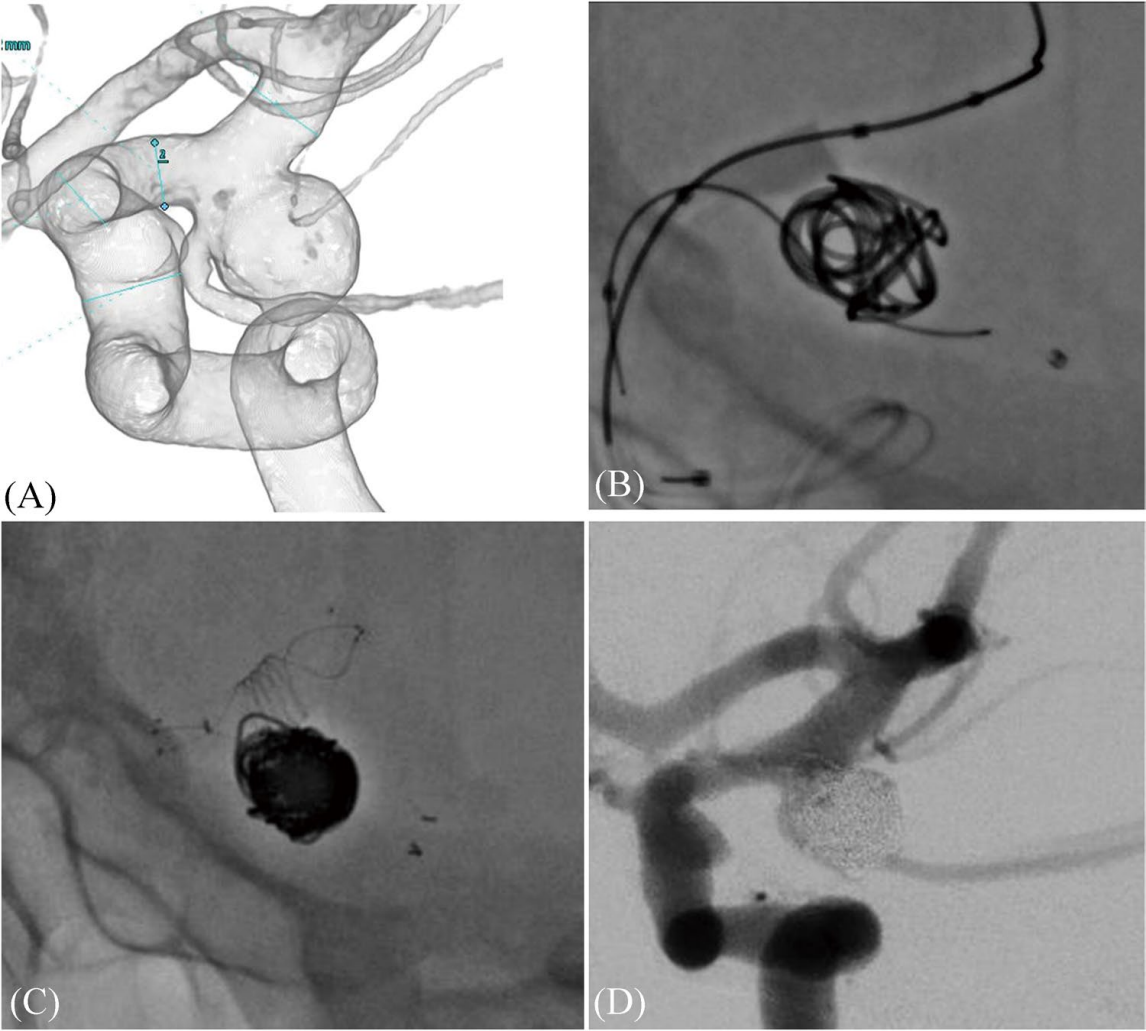
A representative case. A 3D cerebral angiography image demonstrating a right PCOM aneurysm incorporating the orifice of the PCOM (**A**). Using the balloon-assisted technique, coil embolization is performed in the dome of the aneurysm after navigating a microcatheter for stent deployment in the PCOM, preserving the ICA and PCOM (**B**). The ICA and PCOM stents are deployed (**C**). Postoperative angiography shows complete obliteration of the aneurysm, with PCOM preservation (**D**). PCOM, posterior communicating artery; ICA, internal carotid artery

A 45-year-old woman was diagnosed with an unruptured, large, right PCOM aneurysm (patient 3; Tables 1 and 2; Fig. 2A). An 8-Fr guiding catheter (Roadmaster; Goodman, Aichi, Japan) was navigated to the cervical portion of the right ICA via the right femoral artery. First, a balloon catheter (Scepter XC; 4 mm × 11 mm) was positioned in the ICA to cover the aneurysm neck. In addition, a microcatheter for PCOM stenting (Headway 17; MicroVention-Terumo; manually shaped into a micro pigtail) loaded with a micro guide wire (Traxcess 14) was placed in the PCOM without any balloon assistance. Next, a microcatheter to deploy coils was positioned at the aneurysm center. As many coils as possible were inserted using the balloon-assisted technique and catheter assistance of PCOM to avoid the protrusion of coils into the ICA and PCOM (Fig. 2B). The balloon catheter was then removed, and a microcatheter for ICA stenting (Headway 21, MicroVention-Terumo) was navigated into the ICA. The Neuroform Atlas 3 mm × 15 mm was deployed from the PCOM to the distal part of the neck of the aneurysm, and LVIS 4 mm × 22 mm was deployed to cover the aneurysm neck and crushed the PCOM stent between the ICA stent and the coil mass (Fig. 2C). Headway 21 was exchanged with the 7 mm × 7 mm SHOURYU for in-stent percutaneous transluminal angioplasty with an exchange wire to deploy the ICA stent. Subsequently, in-stent percutaneous transluminal angioplasty was performed for complete stent apposition. A packing density of 30.1% and complete obliteration of the aneurysm were achieved without any symptomatic complications (Fig. 2D). Angiographic findings revealed no recurrence 18 months after the procedure.

### Case 2

**Fig. 3:**
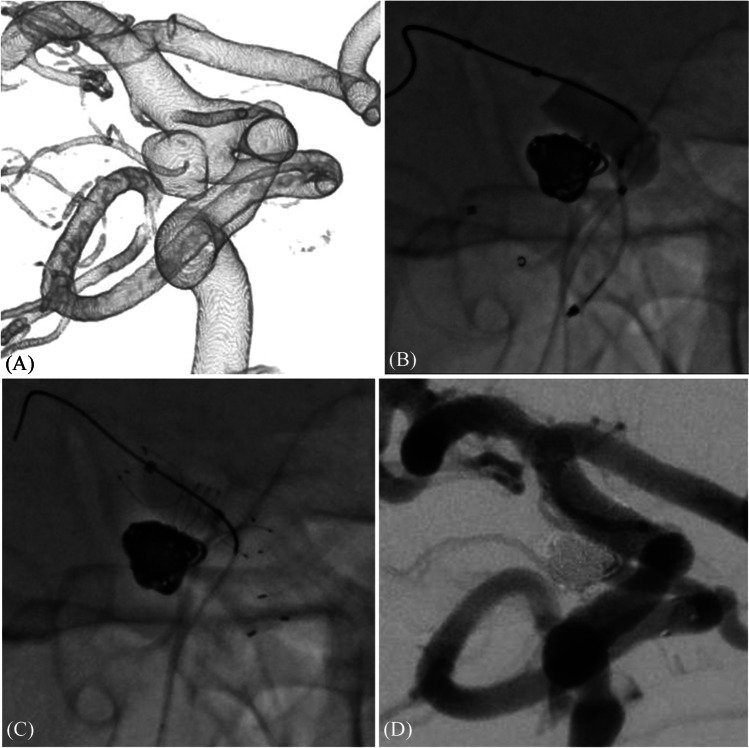
A representative case. A 3D cerebral angiography image demonstrating a right PCOM aneurysm incorporating the orifice of the PCOM (**A**). Using the balloon-assisted technique, coil embolization is performed in the dome of aneurysm after navigating a microcatheter for stent deployment in the PCOM, preserving the ICA and PCOM (**B**). In-stent percutaneous transluminal angioplasty is performed for complete stent apposition after the ICA and PCOM stents are deployed (**C**). Postoperative angiography shows complete obliteration of the aneurysm, with PCOM preservation (**D**). PCOM, posterior communicating artery; ICA, internal carotid artery

A 48-year-old man was diagnosed with an unruptured right PCOM aneurysm (patient 7; Tables 1 and 2; Fig. 3A). An 8-Fr guiding catheter (Roadmaster) was navigated to the cervical portion. First, a balloon catheter (Scepter C; 4 mm × 15 mm) was positioned in the ICA to cover the aneurysm neck. In addition, a microcatheter for PCOM stenting (SL10 pre-shaped J), loaded with a micro guide wire (Tenrou 1014), was placed in the PCOM with balloon assistance. Next, balloon-assisted coiling of the aneurysm was performed (Fig. 3B). The balloon catheter was removed, and a microcatheter for ICA stenting (Headway 21) was navigated into the ICA. In advance, ICA stent as LVIS 4 × 17 mm was partially deployed at the ICA distal to the aneurysm to fix the distal end of the ICA stent. Thereafter, the Neuroform Atlas 3 × 15 mm was deployed from the PCOM to the distal part of the neck of the aneurysm, and the LVIS was fully deployed to cover the aneurysm neck and crushed the PCOM stent between the ICA stent and the coil mass. In-stent percutaneous transluminal angioplasty was performed for complete stent apposition with SHOURYU 7 × 7 mm (Fig. 3C). A packing density of 38.2% and complete obliteration of the aneurysm were achieved without any symptomatic complications (Fig. 3D). Angiographic findings revealed no recurrence after 16 months. The operative technique performed for the illustrative case of patient 7 is presented in Supplemental Video.

## Discussion

### Principal of λ stenting

The present study describes the safety and efficacy of a newly developed stenting method called the “λ stenting technique” for treating PCOM aneurysms with fetal-type PCOM originating from the aneurysm dome. The λ stenting technique is characterized by crushing the PCOM stent between the ICA stent and a coil mesh. In the field of coronary intervention, the crush technique has been widely used for coverage of the bifurcation, including the side branch ostium, to prevent restenosis at the ostium of the side branch in the gap between the main branch and side branch stents [9]. Similarly, the λ stenting technique is proposed herein for secure coverage of the ostium of the PCOM without a gap between the ICA and PCOM, as the gap often becomes problematic for the below-stated T configuration stenting technique. Therefore, the λ stenting technique can induce high packing density for fetal-type PCOM aneurysms, even for those that have the ostium of the PCOM at their dome. In addition, the λ stenting technique, which is a multilayer sealing of the aneurysm neck with one ICA stent mesh and two crushed stent meshes, may result in high metal coverage at the neck, leading to a flow diversion effect. High packing density has been reported to lead to a reduced aneurysm recurrence [10]. Therefore, the λ stenting technique could safely achieve a low recurrence rate for fetal-type PCOM aneurysms without increasing perioperative complications.

### λ stenting versus other stenting techniques and direct surgery

**Fig. 4:**
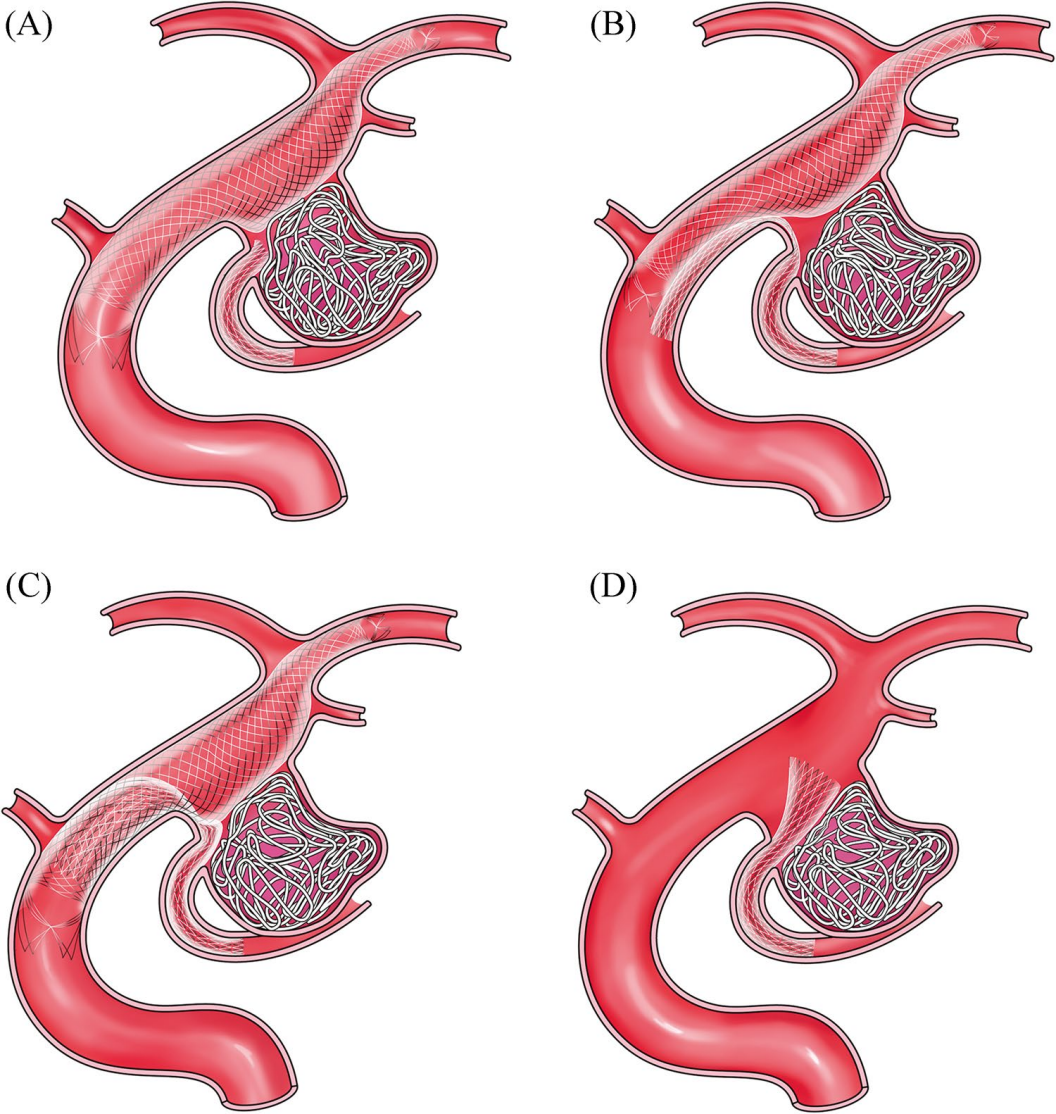
Schematic image of the various complex stenting techniques for fetal-type PCOM aneurysms. T configuration stenting (**A**), Y configuration stenting (kissing and crossing; **B** and **C**, respectively) and PCOM stenting (**D**) are depicted. PCOM, posterior communicating artery

Various complex stenting techniques for endovascular treatment of fetal-type PCOM aneurysms have been reported previously (Fig. 4) [11–13]. The present study indicates that the λ stenting technique may be more effective than other stenting techniques. In PCOM stenting alone, it is difficult to definitively preserve the ICA because PCOM stenting does not cover the aneurysm neck. In the T configuration stenting technique, it is difficult to definitively preserve the PCOM because the occurrence of gaps between the ICA and PCOM stenting cannot be completely eliminated. Accordingly, in these techniques, tight packing of aneurysms is difficult for definite preservation of the ICA and PCOM. Alternatively, in the λ stenting technique, it is possible to definitively preserve the ICA and PCOM because both the ICA and PCOM stent meshes can protect the relevant arteries by utilizing the crush technique.

Flow diverters have been increasingly used in endovascular treatment of PCOM aneurysms as well as wide-necked large and giant aneurysms. Several reports have demonstrated that flow diverters, such as the Pipeline Embolization Device (PED; Medtronic, Minneapolis, Minnesota), contribute to the effective rate of occlusion but carry a risk of PCOM occlusion [6, 14, 15]. PCOM occlusion following flow diverter deployment has not caused any neurological symptoms [6, 14, 15]. However, flow diverter treatment for fetal-type PCOM aneurysms has been associated with incomplete aneurysm occlusion [6, 7]. Therefore, endovascular coiling or surgical clipping is recommended for fetal-type PCOM aneurysms.

Surgical clipping has been considered beneficial for fetal-type PCOM aneurysms [4]. However, fetal-type PCOM aneurysms carry a greater risk of periprocedural ischemic injury even with surgical clipping [16]. Reconstruction clipping of the fetal-type PCOM aneurysm originating from the dome is even difficult when attempting to achieve complete obliteration. Although a superficial temporal artery and posterior cerebral artery bypass are performed, followed by complete aneurysm clipping that involves sacrificing the PCOM, a PCOM perforator infarction can be a valid concern. Furthermore, anatomical features of aneurysms, such as the adherent anterior choroidal artery, projection of aneurysms, and the surgical site, could cause surgical clipping-related complications [17–19]. In contrast, the λ stenting technique could safely achieve a high packing density for the fetal-type PCOM aneurysms because the aforementioned anatomical features never influence the results of endovascular treatment.

### Technical tips for the endovascular procedure

Catheterization of the PCOM arising from the dome of the aneurysm with a steep angle can be a difficult procedure. The order of PCOM catheterization, coiling, and stent deployment is variable in the clinical setting. The order of these procedures depends on the difficulty of PCOM catheterization due to a patient’s unique vascular anatomy, such as tortuosity of the access route and orifice of the PCOM angle. In most cases, the PCOM catheter could be introduced with/without balloon assistance having been previously placed in the ICA. In addition, in cases where primary PCOM cannulation is difficult, adjuvant techniques can be applied, such as half deployment of the ICA stent before PCOM cannulation. Accordingly, ICA stenting and balloon assistance could help PCOM cannulation by acting as a scaffold for microcatheters. For PCOM cannulation, we applied a pre-shaped microcatheter or a simple manual shape, such as a micro pigtail, for cannulation of the PCOM according to the anatomical variation. These shaped microcatheters enabled a steerable micro guidewire with a soft tip to directly select the PCOM without scratching the aneurysm surface.

To prevent PCOM stent-edge placement of the distal neck of the aneurysm from unintentionally migrating toward the aneurysm dome, we always prepare a coil frame as a scaffold with balloon assistance before PCOM stenting. Further, to deploy the PCOM stent in opening the distal flair forward distal neck of the aneurysms, a microcatheter for the PCOM stent needs to be slightly compressed when distal flair is deployed.

## Limitations

A major drawback of the λ stenting technique appears to be thromboembolic complications caused by the complex stenting procedure. Complex stenting techniques, such as Y-configuration stenting, have been reported to cause thromboembolic complications, but are considered to be acceptable [11, 12]. The mesh coverage of the PCOM orifice in λ stenting is almost the same as other complex stenting and flow diverter placement. Moreover, the metal surface exposed in the ICA flow is reduced compared to Y-configuration stenting. Hence, λ stenting could be performed without major thromboembolic complications.

The small number of patients and the short follow-up period in this preliminary study may be inadequate to draw definite conclusions on long-term outcomes and adequate maintenance of antiplatelet agents. Therefore, further studies with longer follow-up periods are needed to evaluate these points.

## Conclusions

The λ stenting technique can enable a dense and stable coil packing of PCOM aneurysms with fetal-type PCOM originating from the aneurysm dome, while preserving the PCOM. The λ stenting technique is considered safe and effective, as reported by this study. Therefore, it could become an alternative treatment option for this sub-type of intracranial aneurysms.

## Supplementary Information

ESM 1 (MP4 165 mb)

## Data Availability

Not applicable
